# Association of diabetes-related kidney disease with cardiovascular and non-cardiovascular outcomes: a retrospective cohort study

**DOI:** 10.1186/s12902-019-0417-9

**Published:** 2019-08-27

**Authors:** James B. Wetmore, Suying Li, Thanh G. N. Ton, Yi Peng, Michael K. Hansen, Cheryl Neslusan, Ralph Riley, Jiannong Liu, David T. Gilbertson

**Affiliations:** 1 Chronic Disease Research Group, Hennepin Healthcare Research Institute, 701 Park Avenue, Suite S4.100, Minneapolis, MN 55415 USA; 2 Division of Nephrology, Hennepin Healthcare, Minneapolis, MN USA; 3 Precision Health Economics, Oakland, California USA; 4 Janssen Research & Development, LLC, Spring House, PA USA; 5 Janssen Global Services, LLC, Raritan, NJ USA

**Keywords:** Cardiovascular disease, Diabetes, End-stage renal disease, Kidney disease

## Abstract

**Background:**

Diabetes-related kidney disease is associated with end-stage renal disease and mortality, but opportunities remain to quantify its association with cardiovascular and non-cardiovascular morbidity outcomes.

**Methods:**

We used the Truven Health MarketScan Commercial Claims and Encounters Database, 2010–2014, which includes specific health services records for employees and their dependents from a selection of large employers, health plans, and government and public organizations. We used administrative claims data to quantify the association between diabetes-related kidney disease and end-stage renal disease, myocardial infarction, congestive heart failure, stroke, and infections. Cox proportional hazard regression models were used to estimate adjusted hazard ratios of developing complications.

**Results:**

Among 2.2 million patients with diabetes, 7.1% had diabetes-related kidney disease: 13.5%, stage 1–2; 33.8%, stage 3; 13.2% stages 4–5; 39.5%, unknown stage. In multivariable Cox proportional hazard models adjusted for demographic characteristics, baseline comorbid conditions, and total hospital days during the baseline period, hazard ratios for each outcome increased with greater diabetes-related kidney disease severity (stage 1–2 vs. stage 4–5) compared with no diabetes-related kidney disease: myocardial infarction, 1.2 (95% confidence interval 1.1–1.4) and 3.1 (2.9–3.4); congestive heart failure, 1.7 (1.6–1.9) and 5.6 (5.3–5.8); stroke, 1.3 (1.2–1.5) and 2.3 (2.1–2.5); infection, 1.4 (1.3–1.5) and 2.9 (2.8–3.0). Among patients with stage 4–5 disease, 36-month cumulative incidence was nearly 22.8% for congestive heart failure, and 25.8% for infections.

**Conclusions:**

Diabetes-related kidney disease appears to be formally diagnosed at a more advanced stage than might be expected, given clinical practice guidelines. Risks of cardiovascular and non-cardiovascular outcomes are high.

**Electronic supplementary material:**

The online version of this article (10.1186/s12902-019-0417-9) contains supplementary material, which is available to authorized users.

## Introduction

Type 2 diabetes is one of the cardinal threats to public health, with approximately 422 million individuals affected worldwide as of 2014 [1]. In the US alone, 29.1 million persons, or 9.3% of the population, have diabetes [2]. In addition to being associated with all-cause and cardiovascular mortality [3], diabetes is associated with development of chronic kidney disease (CKD), commonly termed diabetes-related kidney disease (DKD). Of the 120,000 new cases of end-stage renal disease (ESRD) in the US annually, 44% are attributed to diabetes [4].

In patients with type 2 diabetes, the association of kidney disease with mortality has recently been quantified [5]. However, determination of the stages at which DKD is detected and how DKD stage is associated with major cardiovascular and non-cardiovascular events has not, to our knowledge, been quantified in a large administrative dataset. To investigate these questions, we created a large retrospective cohort of patients enrolled in an employer group health plan (EGHP) to calculate incidence and prevalence of DKD and the associated risks of ESRD, myocardial infarction (MI), congestive heart failure (CHF), stroke, and infection. We elected to study infection specifically because of evidence that diabetes is associated with this outcome [6, 7]; as such, we reasoned that it would serve as an informative counterpoint to cardiovascular disease-related outcomes.

We hypothesized that advancing DKD stage would be associated with increasing risk of these outcomes in patients with diabetes and kidney disease. A better understanding of how DKD stage is associated with risks of morbid events, we reasoned, would inform researchers, healthcare providers, policy makers, and other stakeholders about the potential implications of interrupting the development of kidney disease in these patients, and underscore the public health importance of prevention, detection, and treatment of DKD.

## Materials and methods

### Data source

We used the Truven Health MarketScan Commercial Claims and Encounters Database for 2010–2014, which includes specific health services records for employees and their dependents. This database, which derives from a selection of more than 350 large employers, health plans, and government and public organizations, includes information on person-specific clinical utilization, expenditures, and enrollment across inpatient and outpatient services. The database links paid claims and encounter data to detailed patient information across sites and types of providers and over time. These data represent the medical experience of insured employees and their dependents for active employees, early retirees, and Medicare-eligible retirees with employer-provided Medicare Supplemental plans, among others. At the time we undertook the study, 2014 was the most recent year of data available for purchase.

### Study design and cohort creation

The overall approach used to define kidney disease and health outcomes is shown in Additional file 1: Figure S1. Determining the index date of DKD relied on assuring that diabetes preceded DKD. To identify patients with diabetes, claims for 2010 through 2013 were searched using International Classification of Diseases, Ninth Revision, Clinical Modification (ICD-9-CM) diagnosis codes (Additional file 1: Table S1) on one or more inpatient claims or two or more outpatient claims on different dates within 365 days. The diabetes date for patients with qualifying diabetes claims during 2010 was January 1, 2011. The diabetes date for patients with qualifying diabetes claims after January 1, 2011, was defined as the earliest of the discharge date for an inpatient claim or the second outpatient date (since a single outpatient claim might represent “rule-out” diagnostic evaluation). Patients aged younger than 18 years at the diabetes date, or who had less than 1 year of insurance coverage before the diabetes date, were excluded.

Patients with kidney disease were identified among the 2011–2013 diabetes analytic cohort. We operationally assumed that CKD in patients with diabetes represented DKD, which was identified using ICD-9-CM diagnosis codes (Additional file 1: Table S1) on one or more inpatient claims or two or more outpatient claims on different dates within 365 days. DKD was considered prevalent if DKD codes were found in the 1 year prior to the diabetes date, or incident if found after the diabetes date. Among DKD patients, if laboratory data were available, CKD stage was first defined using estimated glomerular filtration rate (eGFR) from the CKD-EPI 2009 equation [8], in which stages 1 to 5 were defined [9]. If laboratory-based information was not available, CKD stage code (585.1–585.5) claims were used. If different CKD stage code claims were available, the code for the highest stage was used. Patients with unknown CKD stage were included in the analysis, but are displayed as a separate group.

### Covariates

To define covariates, we established a 1-year baseline period before the diabetes date for non-DKD patients and a 1-year period before the DKD index date for DKD patients. Comorbid conditions, shown in Additional file 1: Table S1, were attributed if at least one inpatient or two outpatient claims on different days were identified during the baseline period, and were based on the Elixhauser taxonomy [10]. We also included age (treated as a categorical variable, divided into four groups), sex, and total hospital days during the baseline period as covariates; information on other potentially important covariates, such as race, smoking history, and body mass index, is not available in the MarketScan dataset.

### Outcomes

Patients were followed from DKD index date until study outcomes, end of insurance coverage, December 31, 2014, or, for patients who developed incident DKD, the incident DKD date (at which point these patients contributed person-time to DKD). Outcomes selected were those that could plausibly be related to, or affected by the presence of, DKD, namely ESRD, MI, CHF, stroke, and infections (codes appear in Additional file 1: Table S1**)**.

### Statistical analysis

The incidence rate of DKD was calculated as number of new cases over the 3 years divided by total diabetes (non-DKD) person-time, expressed as number of cases per 1000 patient-years. The denominator population used to calculate the incidence rate excluded prevalent DKD patients.

Outcome analyses included non-DKD and prevalent and incident DKD patients. Unadjusted rates of the outcome events were calculated as number of events per 1000 patient-years over 3 years of follow-up. Cox proportional hazard regression models were used, and adjusted hazard ratios of developing these complications were estimated for patients with diabetes at different stages of kidney disease compared with non-DKD patients with diabetes, after adjustment for the baseline covariates listed above. The cumulative incidence of each outcome was estimated using the Kaplan-Meier method, and adjusted cumulative incidence probabilities were estimated using the Cole and Hernan method [11].

## Results

Slightly fewer than 3 million individuals were identified as having diabetes in 2011–2013, insurance coverage necessary for full observability, and no ESRD; of these, slightly more than 2.2 million were aged 18–64 years, and they comprised the analytic sample. Details of the creation of the analytic cohort comprising incident and prevalent diabetes patients are shown in Additional file 1: Figure S2. Of all patients with diabetes, 157,196 (7.1%) had DKD. Of patients with DKD, 21,212 (13.5%) were at stage 1–2, 53,074 (33.8%) were at stage 3, and 20,814 (13.2%) were at stage 4–5; for 62,096 (39.5%), CKD stage was missing.

Baseline demographic and comorbidity information is shown in Table 1. Individuals with DKD were older than those with diabetes and no kidney disease, but we found no clear trend of increasing age with kidney disease stage. Men comprised more than half the cohort, regardless of DKD presence or disease stage. Patients with the greatest comorbidity burden (as measured by four or more conditions) made up an increasingly larger fraction of the cohort as stage of kidney disease increased.

**Table 1:** Baseline demographic characteristics and major comorbid conditions among patients with diabetes, by stage of kidney disease

	Non-DKD	Stage of Kidney Disease			
		1–2	3	4–5	Missing/Unknown
*N*	2,056,738	21,212	53,074	20,814	62,096
Mean age (SD), years	52.9 (9.4)	54.8 (8.4)	57.7 (6.6)	56.7 (7.3)	54.6 (8.7)
Age groups, years, %					
18–44	18.6	13.0	5.7	8.1	13.6
45–54	31.5	28.3	20.4	23.4	27.6
55–59	24.0	25.8	27.3	26.8	25.6
60–64	26.0	32.9	46.7	41.7	33.2
Sex, %					
Male	53.5	59.8	58.9	57.5	58.7
Female	46.5	40.2	41.1	42.5	41.3
Hospital stays (SD), days	0.6 (4.0)	2.0 (7.4)	3.2 (9.8)	5.4 (13.2)	3.3 (10.9)
Number of comorbid conditions, %					
0	17.4	1.9	0.9	1.0	1.8
1	30.4	10.7	6.8	5.5	12.9
2	29.5	22.3	17.9	13.0	23.8
3	13.0	22.6	20.1	15.7	21.2
≥ 4	9.8	42.6	54.4	64.8	40.3
Selected comorbid conditions, %					
Congestive heart failure	2.0	6.9	13.6	21.3	9.0
Valvular disease	1.4	3.5	5.5	7.7	4.2
Cardiac arrhythmias	3.8	8.5	12.7	15.0	10.4
Hypertension, uncomplicated	44.1	67.2	72.8	72.8	61.5
Hypertension, complicated	1.7	16.2	23.9	34.3	12.2
Peripheral vascular disorders	1.6	4.8	7.1	9.7	5.3
Pulmonary circulation disorders	0.6	1.9	3.2	4.2	2.6
Chronic pulmonary disease	5.8	9.7	12.1	13.4	10.5
Liver disease	2.1	4.2	4.3	5.9	4.9
Peptic ulcer disease excluding bleeding	0.2	0.5	0.5	0.7	0.5
Blood loss anemia	0.3	0.7	1.0	1.7	0.8
Deficiency anemia	1.6	4.3	5.9	9.0	3.7
Coagulopathy	0.7	1.9	2.8	3.9	2.6
Stroke	1.6	4.2	6.1	7.6	4.8
Paralysis	0.3	0.7	0.9	1.2	1.0
Other neurological disorder	1.2	2.6	3.3	4.9	3.4
Hypothyroidism	7.9	9.9	11.6	10.7	10.7
Fluid and electrolyte disorders	2.8	11.4	16.3	25.4	14.6
RA/collagen vascular disease	1.9	3.6	4.0	3.7	3.0
AIDS/HIV	0.2	0.3	0.4	0.4	0.3
Lymphoma	0.3	0.6	0.9	1.2	0.8
Solid tumor without metastasis	3.0	4.7	6.5	6.0	5.0
Metastatic cancer	0.4	0.7	1.3	1.4	1.3
Obesity	6.5	12.8	13.3	12.9	13.2
Weight loss	0.6	1.4	1.9	3.3	2.0
Depression	6.4	8.3	9.3	8.4	9.7
Psychoses	0.4	0.9	1.2	1.5	1.2
Alcohol abuse	0.6	1.1	1.1	1.7	1.5
Drug abuse	0.4	0.8	0.8	1.0	1.1

*Note:* Due to rounding, some column percentage totals may slightly exceed 100%

*CKD* Chronic kidney disease, *DKD* Diabetes-related kidney disease, *RA* Rheumatoid arthritis, *SD* Standard deviation

The DKD incidence rate was 23.4 (95% confidence interval [CI], 23.2–23.5) per 1000 patient-years. The rate was higher for men (25.8, 25.6–26.1) than for women (20.6, 20.4–20.8). The distribution of first observed DKD stage was 14.1% at stage 1–2, 33.8% at stage 3, 6.8% at stage 4, 5.4% at stage 5, and 39.9% at unknown stage. Among patients with known stage of kidney disease when DKD was diagnosed, therefore, 76.5% were at stage 3 or later. Percentages differed little by sex. Distribution of kidney disease stage at diagnosis is shown in Additional file 1: Table S2.

Unadjusted event rates, per 1000 patient-years, for ESRD, MI, CHF, stroke, and infection for patients with known DKD stage are shown in Table 2 over 3 years of follow-up. Event rates increased, in some cases sharply, for all events as stage of kidney disease increased (worsened). This was true for all ages, both sexes, and all comorbidity levels. As would be expected for ESRD, the overall rate was higher, at 152.8, for patients at stages 4–5 compared with the rate for patients at stages 1–2 (4.0); for comparison, the rate for individuals without DKD was 0.5. Rates of CHF were also higher for patients at stages 4–5 (199.9 versus 28.4 for stages 1–2). Rates of infection were much higher as DKD advanced (179.6 for stages 4–5; 50.1 for stages 1–2). In all cases, event rates for patients with DKD of unknown stage were between those for stage 1–2 and stage 3.

**Table 2:** Unadjusted events rate (per 1000 patient-years) by stage of chronic kidney disease among patients with diabetes, 2011–2013

	ESRD				MI				CHF			
	CKD Stage				CKD Stage				CKD Stage			
	Non-DKD	1–2	3	4–5	Non-DKD	1–2	3	4–5	Non-DKD	1–2	3	4–5
Overall	0.5	4.0	13.1	152.8	4.6	7.6	15.3	32.2	7.1	28.4	74.6	199.9
Ages, yrs., %												
18–44	0.3	5.0	23.9	252.1	1.9	4.2	10.2	18.1	2.8	17.0	70.7	148.3
45–54	0.4	4.0	16.7	179.0	4.3	6.9	15.5	30.4	5.3	23.8	71.9	186.9
55–59	0.6	3.5	12.2	142.6	5.4	7.4	14.8	31.0	8.2	26.2	69.0	199.5
60–64	0.8	3.9	10.6	127.2	6.3	9.9	16.2	37.0	11.7	39.4	80.0	218.3
Sex												
Male	0.6	4.5	14.3	165.9	5.9	8.6	16.7	32.6	7.8	31.5	80.4	206.0
Female	0.4	3.2	11.4	135.7	3.2	6.2	13.4	31.7	6.4	23.9	66.3	191.7
No. comorbid conditions												
0	0.4	0.0	5.3	172.9	4.5	8.4	4.0	32.8	3.5	10.0	11.9	92.6
1	0.3	1.1	3.7	137.4	3.7	5.3	11.1	19.7	3.3	3.5	21.0	90.5
2	0.4	1.6	4.0	133.2	4.2	5.1	8.4	22.1	4.6	7.6	16.6	98.0
3	0.6	3.3	7.7	133.1	5.2	5.3	10.3	23.6	9.1	11.7	26.7	111.8
≥ 4	1.8	6.7	20.0	164.4	9.0	11.0	20.7	38.3	35.2	57.5	123.7	261.0
	Stroke				Infection							
	Non-DKD	1–2	3	4–5	Non-DKD	1–2	3	4–5				
Overall	4.6	9.0	14.5	25.2	22.7	50.1	84.5	179.6				
Ages, yrs., %												
18–44	1.7	5.2	9.4	23.6	20.6	59.9	95.8	201.0				
45–54	3.5	8.1	14.4	26.3	20.0	47.6	91.7	184.5				
55–59	5.4	9.7	14.1	25.0	23.5	45.8	77.1	167.3				
60–64	7.3	10.8	15.5	25.1	27.2	52.1	84.5	181.0				
Sex												
Male	5.0	8.3	14.6	25.3	20.7	43.0	80.0	168.6				
Female	4.2	9.9	14.3	25.1	25.0	60.6	91.0	194.5				
No. comorbid conditions												
0	3.8	3.3	10.6	11.9	14.6	21.7	38.3	59.7				
1	3.2	3.5	9.1	20.1	14.5	15.5	34.3	99.5				
2	3.9	5.6	7.5	21.0	17.8	26.5	33.3	96.2				
3	5.5	6.7	9.3	20.5	29.7	31.7	43.0	112.8				
≥ 4	12.6	14.0	20.0	28.3	77.9	85.0	127.8	227.7				

*CHF* Congestive heart failure, *CKD* Chronic kidney disease, *ESRD* End-stage renal disease MI, acute myocardial infarction

**Fig. 1:**
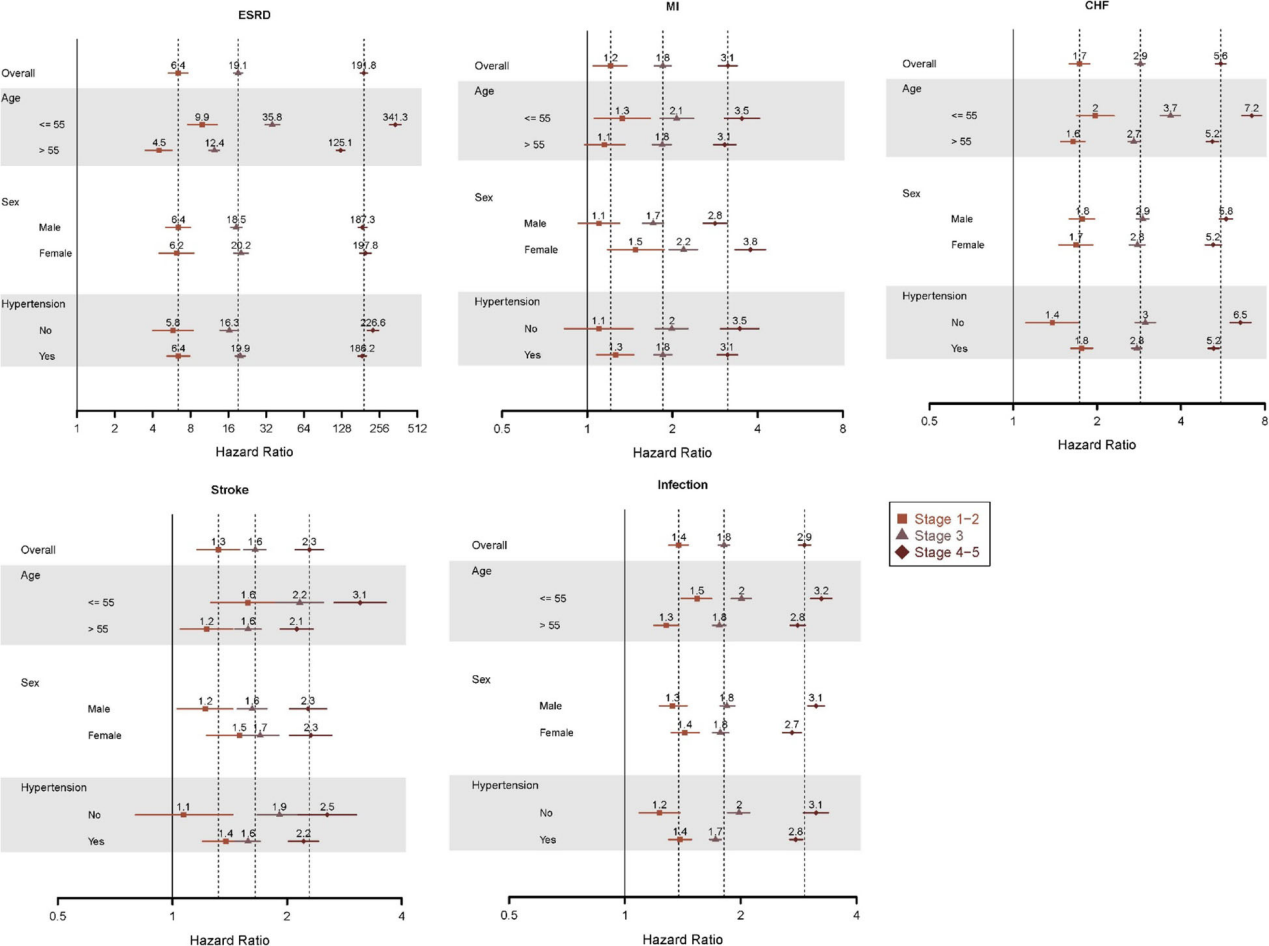
Adjusted hazard ratios for key morbidity outcomes, by stage of CKD. CHF, congested heart failure; CKD, chronic kidney disease; ESRD, end-stage renal disease; MI, myocardial infarction

Adjusted hazard ratios (HRs) are shown in Fig. 1, overall and by strata of age, sex, and presence of hypertension, thereby facilitating comparisons by stage of kidney disease for each outcome. Overall, compared with patients without DKD, HRs of ESRD were 6.4 (95% CI 5.3–7.6) for stages 1–2, 6.4 (5.3–7.9) for stage 3, and 191.8 (180.2–204.2) for stages 4–5. For MI, the corresponding HRs were 1.2 (1.1–1.4), 1.8 (1.7–2.0), and 3.1 (2.9–3.4); for CHF, 1.7 (1.6–1.9), 2.9 (2.7–3.0), and 5.6 (5.3–5.8); for stroke 1.3 (1.2–1.5), 1.6 (1.5–1.8), and 2.3 (2.1–2.5); and infection, 1.4 (1.3–1.5), 1.8 (1.7–1.9), and 2.9 (2.8–3.0) for stage 4–5. Across the spectrum of CKD, risk of ESRD, MI, and stroke were generally higher for younger patients and for women, while risk of heart failure was generally higher for younger patients and for men. Infection risk appeared to be higher only for younger patients.

**Fig. 2:**
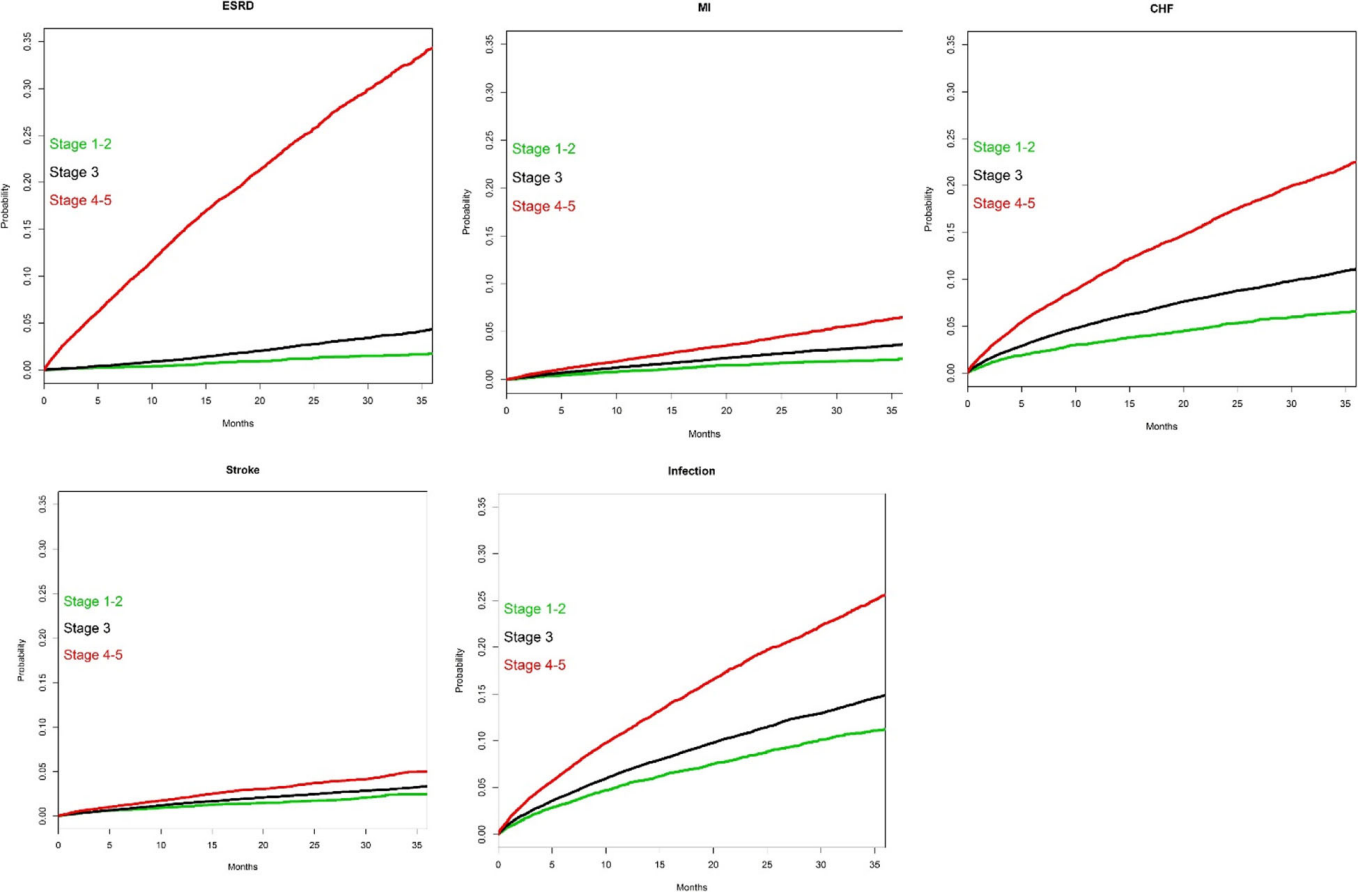
Fully adjusted cumulative incidence curves. CHF, congestive heart failure; ESRD, end-stage renal disease; MI, myocardial infarction

Fully adjusted cumulative incidence curves for patients with known DKD stage are shown in Fig. 2, demonstrating the increased risk of all outcome events over time as CKD stage increases. At 2 years, for example, percentages reaching ESRD were 1.2% for CKD stage 1–2 patients, 2.7% for stage 3, and 25.2% for stage 4–5. Analogous 2-year percentages, by CKD stage, were 1.7, 2.7, and 4.3% for MI; 5.3, 8.6, and 17.2% for CHF; 1.7, 2.4, and 3.6% for stroke; and 8.6, 11.3, and 19.3% for infection.

**Fig. 3:**
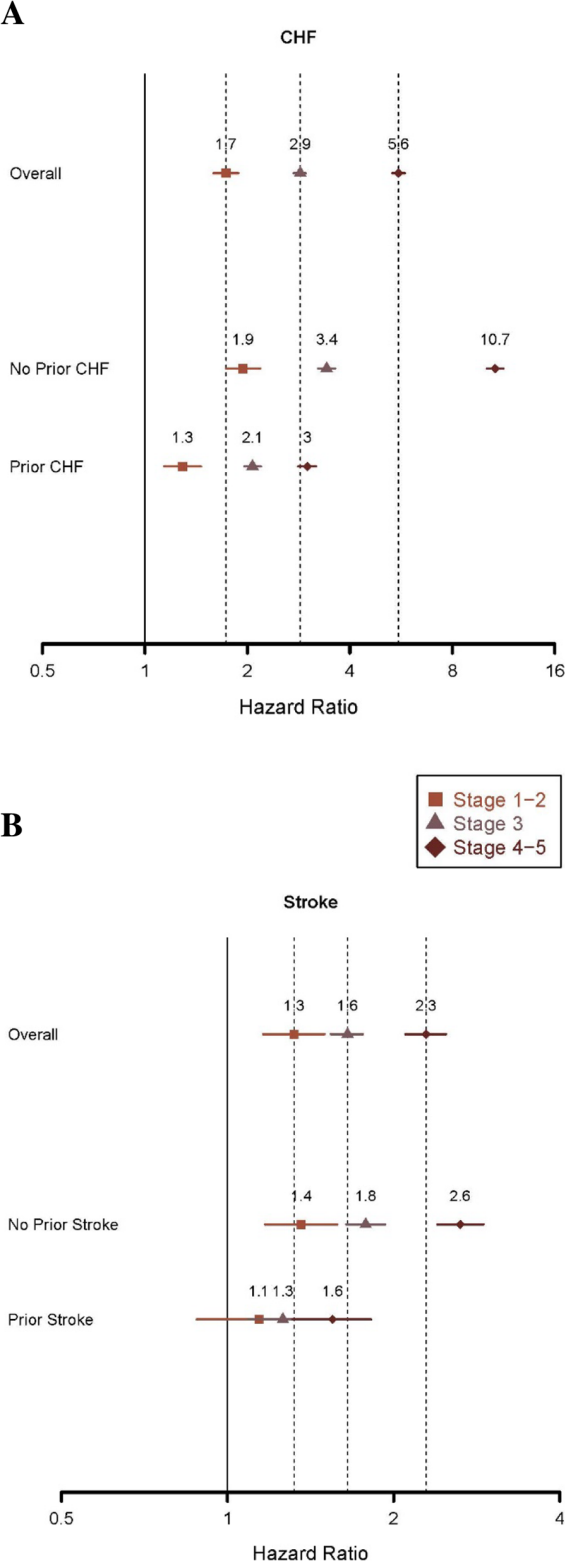
Hazard ratios for CHF (panel A) and stroke (panel B), overall and by presence of preexisting condition. CHF, congestive heart failure

To further explore the association of DKD with outcomes, we sought to understand how the explicit consideration of presence of preexisting CHF and stroke might have altered the results. We performed separate subgroup analysis for patients with CHF (versus without) and with a history of a stroke (versus without). Results, shown in Fig. 3, suggest that while history of CHF and stroke were, as would be expected, associated with future CHF and stroke (respectively) in DKD patients, worsening DKD stage was associated with substantially increased risk of these outcomes.

## Discussion

Using a contemporary sample comprising more than 2.2 million patients with diabetes, we found that more than 3 out of 4 patients identified as having DKD were at stage 3 or higher at diagnosis, suggesting that formal identification occurs most often in patients with lower eGFR, well after pathophysiologic changes are likely to have begun in the kidney. We also found, not unexpectedly, that rates of major outcomes increased substantially as stage of kidney disease worsened. Our findings suggest that prevention of DKD and its progression in patients with diabetes would likely have major population-wide benefits in the form of reduced cardiovascular and non-cardiovascular events.

One contribution of our study is quantification of the stage at which DKD patients are identified, an issue of major public health importance. In the general population, CKD is most commonly diagnosed at stage 3. However, our finding that three-quarters of DKD patients who have relatively ready access to healthcare via private insurance are also identified at stage 3 disease or later is particularly troubling, The American Diabetes Association recommends that all patients with diabetes undergo yearly measurement of serum creatinine (and urinary albumin-to-creatinine ratio) [12, 13] in an attempt detect CKD relatively early in the disease process. While it is possible that patients with earlier stages of kidney disease (e.g., stages 1–2) may be clinically recognized by a healthcare provider as having DKD despite the absence of a code, presence of a code suggests that the disease has been formally recognized. Formal recognition of a condition via the generation of a code is important for automated electronic health system algorithms, which generate a list of a patient’s conditions in the medical record and which often bring automated risk-management strategies (such as “pop-up warnings”) to providers’ attention in real-time. Our results suggest that, even in a population with relatively good access to medical care, many patients appear to be identified comparatively late in the kidney disease process. If correct, this represents a potential missed opportunity to alter the course of DKD, and suggests that both improved implementation of established screening approaches and introduction of novel strategies, such as use of biomarkers [14], may identify the renal ramifications of diabetes earlier than would otherwise be the case.

The other major contribution of our study is quantification of the association of DKD with key clinical outcomes, in contrast to other studies focusing on the costs associated with DKD and its progression [15–18]. Other studies, principally involving Chinese patients, have contributed important estimates of development of diabetes-related complications such as cardiovascular and coronary heart disease, stroke, heart failure, and ESRD, and therefore have reported outcomes based on the timing of diabetes onset (rather than DKD onset) [19–24]. Our study, based on the timing of DKD development, found that risks of MI and stroke were about 85 and 65% higher, respectively, in stage 3 disease (relative to no disease); because most prevalent DKD patients are at this stage, these risks likely closely approximate risks of these events in the DKD population, and suggest that preventing progression of DKD might result in substantial reduction of these events. The relationship of CHF with kidney disease stage was even stronger: DKD stage 3 was associated with a nearly 3-fold risk of CHF, and stage 4–5 with a more than 5.5-fold risk. Within 3 years, 20% of patients at stage 4 or 5, and 10% of patients at stage 3, will develop CHF. While an association of CHF with diabetes has long been recognized [25–30], diabetes-related cardiomyopathy is now recognized as a distinct clinical entity that can develop independently of traditional risk factors such as coronary artery disease and hypertension [31], and that can present phenotypically as either systolic or diastolic dysfunction [32]. It is therefore possible that the risk of CHF we identify may represent “traditional” (or non-diabetes-related) heart failure as well as diabetes-related cardiomyopathy.

We were initially uncertain as to the nature of the association between DKD and infection. We found that the risk pattern for kidney disease stage and infection was generally similar to the patterns for MI and stroke. Both diabetes and CKD represent immunocompromised states; as such, patients with diabetes are at greater risk than those without for a wide range of infections [33]. Our findings suggest that preventing or slowing DKD progression may yield benefits beyond those attributable to the cardiovascular system.

Our estimate of diagnosed DKD incidence in diabetes patients, 23 per 1000 patient-years, appears to be broadly concordant with an estimate in a recent meta-analysis by Koye at al [34]. These authors examined 71 studies from 30 countries and estimated that the risk of an eGFR below 60 mL/min/1.73m^2^ was 2–4% per annum (corresponding to 20–40 per 1000 patient-years). They reported that the risk of developing micro- or macroalbuminuria was higher still, at about 8% per year. As is the case with many studies using large administrative databases, our estimate is based primarily on billing claims data, meaning that had laboratory information on albuminuria and eGFR been more widely available, our estimate might have been higher. Additionally, the patient population, comprising mainly privately insured individuals of working age and their dependents, is likely substantially healthier than the general diabetes population. Thus, the burden of cardiovascular and non-cardiovascular events in diabetes patients with kidney disease might be greater than our results would suggest. However, prevalence of kidney disease among patients with diabetes in our study was 7.1%, lower than estimates from studies of other populations such as the National Health and Nutrition Examination Survey [35–37] and the Kidney Early Evaluation Program [38], in which estimates ranged from approximately 19 to 39%. This finding is not unexpected, as the Truven MarketScan EGHP database is enriched with working-age adults with private health insurance. Prevalence of DKD (defined as eGFR < 60 mL/min/1.73m^2^) in a cohort of Italian patients [39] aged younger than 65 years being treated by diabetologists was 6.8%, comparable to our estimate.

Other investigators have attempted to quantify the risk of progression from DKD to ESRD, but due to differences in study design, direct comparisons should be made with caution. In a cohort of more than 6300 Chinese patients with eGFR > 60 mL/min/1.73m^2^ tracked in a diabetes registry, 7.2% developed CKD stage 5 or 5D over a median follow-up period of 13 years [40]. Given that time spent at non-dialysis stage 5 CKD is generally much shorter than time spent receiving dialysis [41] (that is, at stage 5D), this percentage likely approximates the percentage reaching ESRD. These findings are not inconsistent with our estimate of a 1.8% chance of ESRD development, given that our follow-up period, at 3 years, was shorter than the follow-up period available in the study above.

A major challenge in studying kidney disease etiology in large administrative datasets is lack of detailed clinical information, such as that derived from a kidney biopsy. This is a particularly vexing issue in the case of DKD. While studies have shown that perhaps a third of biopsied patients with diabetes have non-diabetes-related kidney disease [42, 43], these findings are derived from case series of patients undergoing real-world clinical care. The vast majority of patients with diabetes and CKD are not biopsied, suggesting that only atypical cases of putative DKD are actually biopsied (and thus reported on in biopsy studies). Because of this, for the purposes of this study, we attributed CKD in patients with diabetes to DKD, but we acknowledge that, in some cases at least, non-diabetes-related kidney disease was likely the primary cause of kidney disease. A more complete understanding of DKD epidemiology awaits more comprehensive biopsy studies.

Our study had several important limitations. As an observational study, our analysis cannot determine causality. Preexisting vascular disease could contribute to kidney disease development in patients with diabetes, while DKD could contribute to cardiovascular events; we suspect both mechanisms are operative. Further, it is unclear whether prevention of DKD advancement would actually result in attenuation of the risks of the outcomes we studied, although this hypothesis is highly plausible. Our study relied primarily on billing claims data, supplemented by laboratory data, and identification of claims is not equivalent to a medical diagnosis. Duration of diabetes, a disease that patients often have for many decades, was unknown, and therefore could not be specifically controlled for in the analysis, nor could age at time of diabetes onset. A longer follow-up period would have been beneficial to strengthen the estimates of the association between exposures and outcomes, but we purchased the most data possible within the study budget (as well as the most recent data available at the time the study commenced).We also can draw no conclusions about the relative importance of kidney disease in patients with diabetes relative to CKD in patients without diabetes, but that was not the goal of our study. Additionally, as we studied only individuals with private health insurance, generalizability is likely compromised beyond a population highly enriched with younger individuals (aged < 65 years), or to a non-US population. As a consequence, however, our analysis may actually underestimate the burden of disease in the general US population. The Truven MarketScan database also lacks information on out-of-hospital death. Death likely constitutes a substantial competing risk for the outcomes we studied. If rates of death are high in patients with diabetes and DKD, this serves to strengthen the case that kidney disease should be diagnosed as early as possible in these patients. Finally, the present study, which was designed to estimate the associations of DKD (versus no kidney disease) in patients with diabetes, does not permit us to make any inferences about kidney disease in patients without diabetes. As such, any findings regarding the association of kidney disease with outcomes is confined to DKD alone. Our study is unique because we have attempted to quantify the risks associated the DKD and morbid events, in contrast to other studies focusing on the costs associated with DKD and its progression.

## Conclusions

In conclusion, we quantified the association between stage of DKD and risk of major morbid events in a large sample of patients. Risks of cardiovascular and non-cardiovascular events increase substantially with increasing stage of kidney disease, suggesting that rates of morbid events might decrease if DKD can be detected more readily and its progression slowed. We also found that DKD appears to be diagnosed at a more advanced stage of disease than might be expected, even in privately insured individuals who presumably have ready access to healthcare. This is a sobering finding given the emphasis afforded by clinical practice guidelines on screening for kidney disease in diabetes patients.

## Additional file

Additional file 1: **Figure S1.** Approach used to define kidney disease and health outcomes in the analytic cohort. CHF, congestive heart failure; DKD, diabetes-related kidney disease; DM diabetes mellitus; ESRD, end-stage renal disease. **Table S1.** International Classification of Diseases, Ninth Revision, Clinical Modification diagnosis codes used to identify diabetes, chronic kidney disease, comorbid conditions, and adverse events. **Figure S2.** Construction of the analytic cohort. DKD, diabetes-related kidney disease; ESRD, end-stage renal disease; HMO, health maintenance organization. **Table S2.** Stage of incident chronic kidney disease in patients with diabetes, ages 18–64 years, 2011–2013. (PDF 1404 kb)

## Data Availability

Data were obtained from the Truven Health MarketScan Commercial Claims and Encounters Database.

## Abbreviations

ADA: American Diabetes Association
CHF: Congestive heart failure
CKD: Chronic kidney disease
DKD: Diabetes-related kidney disease
eGFR: estimated glomerular filtration rate
EGHP: Employer group health plan
ESRD: End-stage renal disease
HR: Hazard ratio
ICD-9-CM: International Classification of Diseases, Ninth Revision, Clinical Modification
MI: Myocardial infarction
