# Supplementary material for: Association of diabetes-related kidney disease with cardiovascular and non-cardiovascular outcomes: a retrospective cohort study
Source: BMC Endocr Disord. 2019 Aug 27;19:89. doi: 10.1186/s12902-019-0417-9 (PMC6712860; doi:10.1186/s12902-019-0417-9)
Supplement: Supplementary file 1 — Figure S1. Approach used to define kidney disease and health outcomes in the analytic cohort. CHF, congestive heart failure; DKD, diabetes-related kidney disease; DM diabetes mellitus; ESRD, end-stage renal disease. Table S1. International Classification of Diseases, Ninth Revision, Clinical Modification diagnosis codes used to identify diabetes, chronic kidney disease, comorbid conditions, and adverse events. Figure S2. Construction of the analytic cohort. DKD, diabetes-related kidney disease; ESRD, end-stage renal disease; HMO, health maintenance organization. Table S2. Stage of incident chronic kidney disease in patients with diabetes, ages 18–64 years, 2011–2013. (PDF 1404 kb) [file 12902_2019_417_MOESM1_ESM.pdf]

Supplementary Material

Figure S1. Approach used to define kidney disease and health outcomes in the analytic cohort. CHF, congestive heart failure; DKD, diabetic kidney disease; DM diabetes mellitus; ESRD, end-stage renal disease.

Table S1. International Classification of Diseases, Ninth Revision, Clinical Modification diagnosis codes used to identify diabetes, chronic kidney disease, comorbid conditions, and adverse events

Figure S2. Construction of the analytic cohort. DKD, diabetic kidney disease; ESRD, end-stage renal disease; HMO, health maintenance organization.

Table S2. Stage of incident chronic kidney disease in patients with diabetes, ages 18-64 years, 2011-2013.

Figure S1. Approach used to define kidney disease and health outcomes in the analytic cohort. CHF, congestive heart failure; DKD diabetic kidney disease; DM, diabetes mellitus; ESRD, end-stage renal disease.

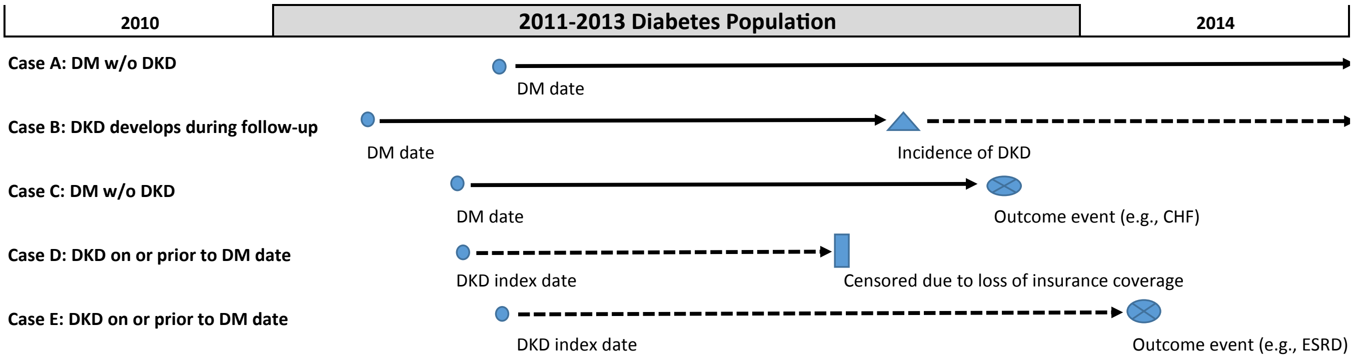

Case A: DM without DKD on or prior to DM date

(1) No incidence of DKD or any outcome event during follow-up

Case B: DM without DKD on or prior to DM date

(1) Incidence of DKD during follow-up
(2) DM contribution time is from the DM date to the DKD incident date
(3) DKD contribution time is from the DKD incident date +1 until end of follow-up or date of outcome event (e.g., CHF)

Note: this patient will appear in two patient groups

(a) DM without DKD (from DM date to incidence of DKD)
(b) DM with DKD (from incidence of DKD to end of follow-up or outcome event date)

Case C: DM without DKD on or prior to DM date

(1) Outcome event during follow-up
(2) DM contribution time is from the DM date to the outcome event date

Case D: Both DM and DKD established on the DKD index date

(1) No outcome event during follow-up

Case E: Both DM and DKD established on the DKD index date

(1) Outcome event during follow-up

Table S1. International Classification of Diseases, Ninth Revision, Clinical Modification diagnosis codes used to identify diabetes, chronic kidney disease, comorbid conditions, and adverse events

Conditions and Events	ICD-9-CM Diagnosis Codes
**Diseases**	
Diabetes	249.4; 250; 357.2; 362.0x; 366.41
CKD	250.4; 581; 583.81; 585-587; 588.0; 588.9; 794.4
**Comorbid conditions**	ICD-9-CM
CHF	398.91, 402.11, 402.91, 404.11, 404.13, 404.91, 404.93, 428.x
Cardiac arrhythmias	426.10, 426.11, 426.13,426.2-426.53, 426.6-426.8,427.0, 427.2, 427.31,427.60, 427.9, 785.0,V45.0, V53.3
Valvular disease	093.2, 394.0-397.1, 424.0-424.91, 746.3-746.6,V42.2, V43.3
Pulmonary circulation disorders	416.x, 417.9
Peripheral vascular disorders	440.x, 441.2, 441.4, 441.7,441.9, 443.1- 443.9, 447.1,557.1, 557.9, V43.4
Hypertension, uncomplicated	401.1, 401.9
Hypertension, complicated	402.10, 402.90, 404.10,404.90, 405.1, 405.9
Paralysis	342.0. 342.1, 342.9-344.x
Other neurological disorders	331.9, 332.0, 333.4, 333.5,334.x, 335.x, 340.x, 341.1-341.9, 345.0, 345.1, 345.4,345.5, 345.8, 345.9, 348.1,348.3, 780.3, 784.3
Chronic pulmonary disease	490-492.8, 493.00-493.91, 494.x-505.x, 506.4
Diabetes, uncomplicated	250.0-250.3
Diabetes, complicated	250.4-250.7, 250.9
Hypothyroidism	243-244.2, 244.8, 244.9
Renal failure	403.11, 403.91, 404.12, 404.92, 585.x, 586.x, V42.0, V45.1, V56.0, V56.8
Liver disease	070.32, 070.33, 070.54, 456.0, 456.1, 456.2, 571.0, 571.2-571.9, 572.3, 572.8, V42.7
Peptic ulcer disease excluding bleeding	531.70, 531.90, 532.70, 532.90, 533.70, 533.90, 534.70, 534.90, V12.71
AIDS/HIV	042.x-044.x
Lymphoma	200.x-202.3x, 202.5-203.0, 203.8, 238.6, 273.3, V10.71, V10.72, V10.79
Metastatic cancer	196.x-199.x
Solid tumor without metastasis	140.x-172.x, 174.x, 175.x, 179.x-195.x, V10.x
RA/collagen vascular disease	701.0, 710.x, 714.x, 720.x, 725.x
Coagulopathy	286.x, 287.1, 287.3-287.5
Obesity	278.0
Weight loss	260.x-263.x
Fluid and electrolyte disorders	276.x
Blood loss anemia	280.0
Deficiency anemia	280.1-281.9, 285.9
Alcohol abuse	291.1, 291.2, 291.5-291.9, 303.9, 305.0, V113
Drug abuse	292.0, 292.82-292.89, 292.9, 304.0, 305.2, 305.9
Psychoses	295.x-298.x, 299.1
Depression	300.4, 301.12, 309.0, 309.1, 311
Stroke	433.x1, 434.x1, 430, 431, 432, 433.x0, 434.x0, 436, 437, 438
**Outcomes**	

ESRD	ICD-9-CM diagnosis codes: 585.6, 585.60, 996.81, V420, V4200; DRG: 008, 302, 512, 652; revenue center codes: 0820-0859
MI*	410.xx (not 410.x2)
CHF*	276.6x, , 402.01, 402.11, 402.91, 404.01, 404.03, 404.11, 404.13, 404.91, 404.93, 428.x
Stroke*	Ischemic stroke: 433.x1, 434.x1; hemorrhagic stroke: 430, 431, 432; cerebrovascular disease: 433.x0, 434.x0, 436
Infection*	036.x, 790.7, 038.x, 041.x, 730.0, 730.1, 730.3-730.9, 391.x, 392.x, 421.x, 422.0, 422.90, 422.92, 480-486, 510.x, 513.x, 590.x, 595.x, 597.x, 599.0, 601.x, 604.0, 608.0, 608.4, 607.1, 607.2, 616.1-616.4, 254.1, 320-324, 382.0-382.4, 383.0-383.2, 478.22, 478.23, 478.24, 478.29, 522.5, 522.7, 527.3, 528.3, 540-542, 566.x, 567.0-567.3, 567.81, 567.9, 569.5, 572.0, 572.1, 575.0, 575.1, 611.0, 614.0-614.5, 615.0, 615.9, 711.0, 997.62, 998.5, 999.3

*Inpatient only, first five positions.

CHF, congestive heart failure; CKD, chronic kidney disease; DRG, diagnosis-related group; ESRD, end-stage renal disease; ICD-9-CM, International Classification of Diseases, Ninth Revision, Clinical Modification; MI, myocardial infarction; RA, rheumatoid arthritis.

Figure S2. Construction of the analytic cohort. DKD, diabetic kidney disease; ESRD, end-stage renal disease; HMO, health maintenance organization.

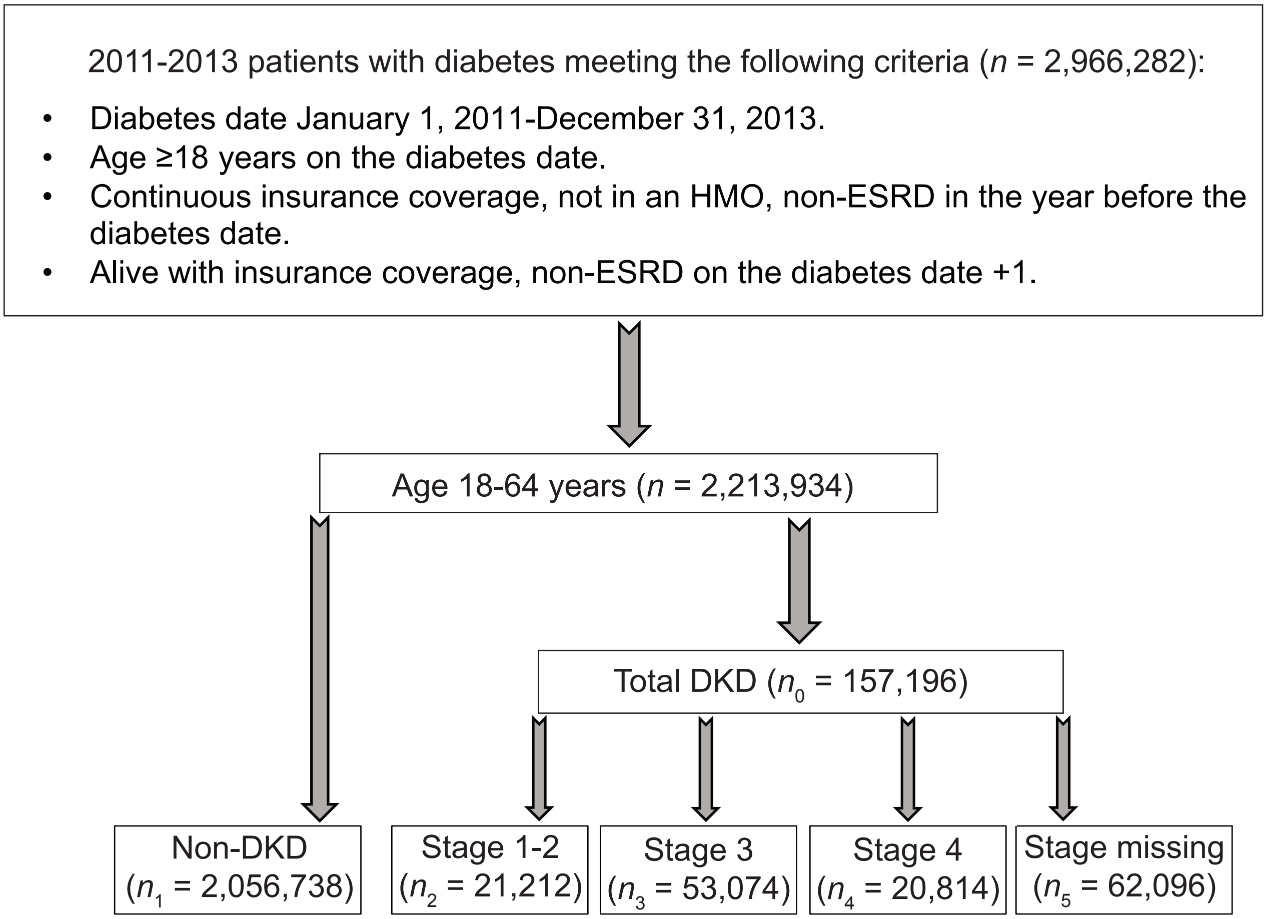

Table S2. Stage of incident chronic kidney disease in patients with diabetes, ages 18-64 years, 2011-2013

Stage	All	Men	Women
All stages, *n*	88,673	51,999	36,674
Stages 1-5, *n*	53,300	31,264	22,036
Stage 1, *n*	4178	2395	2395
% of known stage	7.8	7.7	8.1
% of total	4.7	4.6	4.9
Stage 2, *n*	8368	5126	3242
% of known stage	15.7	16.4	14.7
% of total	9.4	9.9	8.8
Stage 3, *n*	29,940	17,560	12,380
% of known stage	56.2	56.2	56.2
% of total	33.8	33.8	33.8
Stage 4, *n*	6056	3393	2663
% of known stage	11.4	10.9	12.1
% of total	6.8	6.5	7.3
Stage 5, *n*	4758	2790	1968
% of known stage	8.9	8.9	8.9
% of total	5.4	5.4	5.4
Stage unknown, *n*	35,373	20,735	14,638
% of total	39.9	39.9	39.9
